# Synergistic Gas Hydrate and Corrosion Inhibition Using Maleic Anhydride: *N*-Isopropylmethacrylamide Copolymer and Small Thiols

**DOI:** 10.1021/acsomega.3c05828

**Published:** 2023-09-29

**Authors:** Janronel Pomicpic, Malcolm A. Kelland

**Affiliations:** Department of Chemistry, Bioscience and Environmental Engineering, Faculty of Science and Technology, University of Stavanger, N-4036 Stavanger, Norway

## Abstract

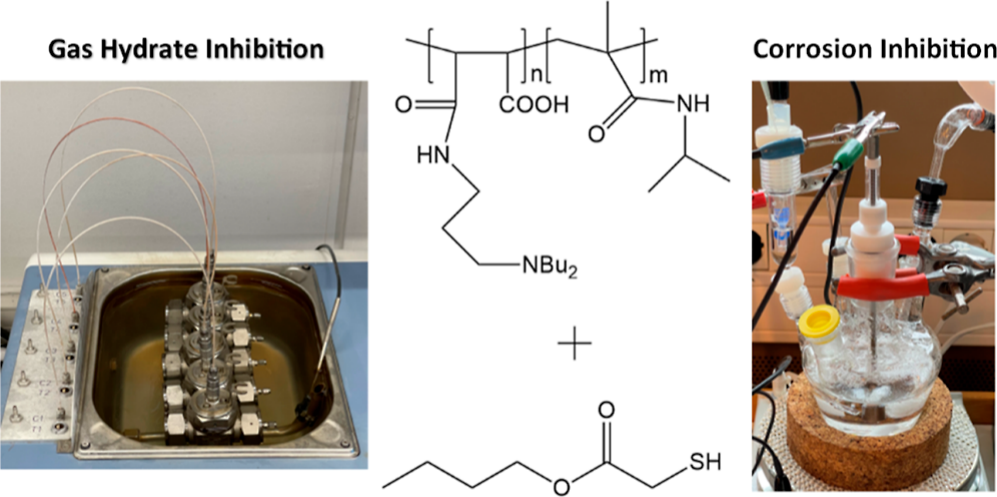

Kinetic gas hydrate inhibitors (KHIs) are often used in combination with film-forming corrosion inhibitors (CIs) in oilfield production flow lines. However, CIs can be antagonistic to KHI performance. In this study, maleic anhydride-*co*-*N*-isopropylmethacrylamide copolymer (MA:NIPMAM) and its derivatives were successfully synthesized and tested for gas hydrate and corrosion inhibition. KHI slow constant cooling (1 °C/h) screening tests in high-pressure rocking cells with synthetic natural gas and CO_2_ corrosion bubble tests in brine were performed in this study. The results revealed that underivatized MA:NIPMAM in water (as maleic acid:NIPMAM copolymer) showed poor KHI performance, probably due to internal hydrogen bonding. However, derivatization of MA:NIPMAM with 3-(dibutylamino)-1-propylamine (DBAPA) to give MA:NIPMAM-DBAPA gave excellent gas hydrate inhibition performance but only weak corrosion inhibition performance. Unlike some KHI polymers, MA:NIPMAM-DBAPA was compatible with a classic fatty acid imidazoline CI, such that neither the KHI polymer performance nor the corrosion inhibition of the imidazoline was affected. Furthermore, excellent dual gas hydrate and corrosion inhibition was also achieved in blends of MA:NIPMAM-DBAPA with small thiol-based molecules. In particular, the addition of butyl thioglycolate not only gave excellent corrosion inhibition efficiency, better than adding the fatty imidazoline, but also enhanced the overall gas hydrate inhibition performance.

## Introduction

In the oil and gas production process, the produced fluids contain hydrocarbons and, oftentimes, substantial amounts of water.^1^ The petroleum industry is mainly concerned with the produced hydrocarbons as these constitute the products of economic interest. It is inevitable, however, to encounter water coproduced with these hydrocarbons. The coproduced water usually contains impurities such as minerals (Ca^2+^and Ba^2+^), dissolved gases (CO_2_ and H_2_S), and microorganisms (bacteria and fungi),^2^ which eventually pose unwanted problems to the production process.^1^ For instance, Ca^2+^ can react with dissolved CO_2_ (in the form of HCO_3_^–^) to precipitate out inorganic compounds called scales.^3^ Also, in conditions of very low temperatures and high pressures, the water molecules themselves entrap some gaseous hydrocarbon molecules to form crystalline compounds called gas hydrates.^4^ Both gas hydrates and scales can accumulate as very hard solids that block the normal flow of the pipelines. A third problem concerning coproduced water is the more familiar process of corrosion. In this case, CO_2_ reacts with water to produce carbonic acid (H_2_CO_3_), which is considered to be a main causative agent for corrosion.^5^ The overall electrochemical process of corrosion is complex as it also involves the biochemical action of microbes that are also present in the produced water. In contrast to gas hydrates and scales, which block the normal flow of pipelines, corrosion causes embrittlement and destroys the pipeline material integrity.^6^

In real oil and gas production operations, problems related to gas hydrates, scales, and corrosion are seldom encountered in isolation. The complex interplay of these production chemistry problems in the field has been reviewed.^7^ Chemical inhibitors are usually the treatment of choice for the prevention of these water-based production problems.^6^ Commercial upstream flowline corrosion inhibitors (CIs) are usually film-forming surfactants although polymeric CIs have also been developed.^8,9^ Hydrate management can be accomplished with several classes of chemicals, one of which is polymeric. Therefore, this opens the possibility of treating hydrates and corrosion with a single polymer.

Recently, there has been increased activity to develop inhibitors that prevent two or more of the water-based production problems (hydrates, corrosion, and scale) in one treatment.^10–14^ Chemicals that have multifunctional inhibition capabilities are more advantageous, especially in fields where these different production problems occur simultaneously.^15–17^ In the development of multifunctional inhibitors, however, three important factors must be considered: effectiveness, compatibility, and cost. The performance of new inhibitors must be comparable or superior to that of existing commercial inhibitors. In addition, there should be no interincompatibilities when mixed with other production chemicals. Lastly, new inhibitors must be economically feasible so that they can be adopted easily by the oil and gas industry.^7^ There has been a great challenge with regard to the development of multifunctional inhibitor formulations. To the best of our knowledge, no single polymer to date has been synthesized that can have gas hydrate, scale, and corrosion inhibiting properties in one macromolecule. Also, incompatibilities may occur when separate gas hydrate, scale, and corrosion inhibitors are mixed together.^18,19^

It has been a goal of our research group to develop new multifunctional inhibitors that have reasonable effectiveness, good compatibility, and low cost. Few chemicals seem to fulfill that role. We have chosen maleic anhydride (MA) and the polymers thereof as the starting points of our experiments. Maleic anhydride is a readily available and cheap monomer that can be homopolymerized to give oligomers or copolymerized with other vinylic monomers to produce a plethora of functional polymers.^20^ The maleic anhydride functional group can be easily derivatized with alcohols or amines in a facile manner to give a wide range of functionality for various oilfield production applications.^21^ Some maleic-based polymers have already been reported to show corrosion inhibition properties.^22,23^ The first development of maleic-based polymers as kinetic hydrate inhibitors (KHIs) was in the mid-1990s by amide-derivatization of maleic anhydride-based copolymers.^24^ Recently, new classes of maleic-based copolymers with improved performance as KHIs were developed by our group.^25–27^ We aim to further extend the applicability of these maleic copolymers to corrosion inhibition as well as scale inhibition.

We started by copolymerizing maleic anhydride with *N*-vinylcaprolactam (VCap) or *N*-isopropylmethacrylamide (NIPMAM) as these are two of the most active and well-known monomer units used in commercial KHI polymers.^6^ The exploration of poly(maleic anhydride-*co*-*N*-vinylcaprolactam) (MA:VCap) and its derivatives for dual gas hydrate and corrosion inhibition has been published with very promising results.^28,29^ Here, we focus on poly(maleic anhydride-*co*-*N*-isopropylmethacrylamide) (MA:NIPMAM) and its derivatives for potential use as dual gas hydrate inhibitor and CI. The use of copolymers of MA with NIPMAM as inhibitors against inorganic scales is beyond the scope of this study and will potentially be reported in a later publication.

We conceived four possible pathways in order to develop a dual-purpose gas hydrate inhibitor and CI based on MA and NIPMAM. One way is to copolymerize with another monomer containing a corrosion-inhibiting functional group. A second way is to copolymerize and then end-cap with a corrosion-inhibiting molecule. A third way is to produce a copolymer of MA and NIPMAM and then use postpolymerization functionalization methods to modify the anhydride group with a corrosion-inhibiting molecule.

The three methods give a single polymer containing all of the gas hydrate- and corrosion-inhibiting functional groups in one macromolecule. The synthetic processes, however, are more challenging, so in this study we opted to follow a fourth pathway, which is to produce copolymers based on MA and NIPMAM and then separately adding a “synergist”, which we designed to enhance the overall gas hydrate inhibition performance and at the same time give good CO_2_ corrosion inhibition. Future reports will explore the use of the other three pathways.

## Experimental Section

### Materials

The chemicals used in the polymer syntheses including maleic anhydride, *N*-isopropylmethacrylamide, 2,2-azobis(2-methylpropionitrile), 1,2-dimethoxyethane, 3-(dibutylamino)-1-propylamine, and hydrogen peroxide were purchased and used as received from Merck. All other chemicals used in this study were purchased from VWR/Avantor or Merck and were used as received.

### Polymer Syntheses

**Figure 1:**
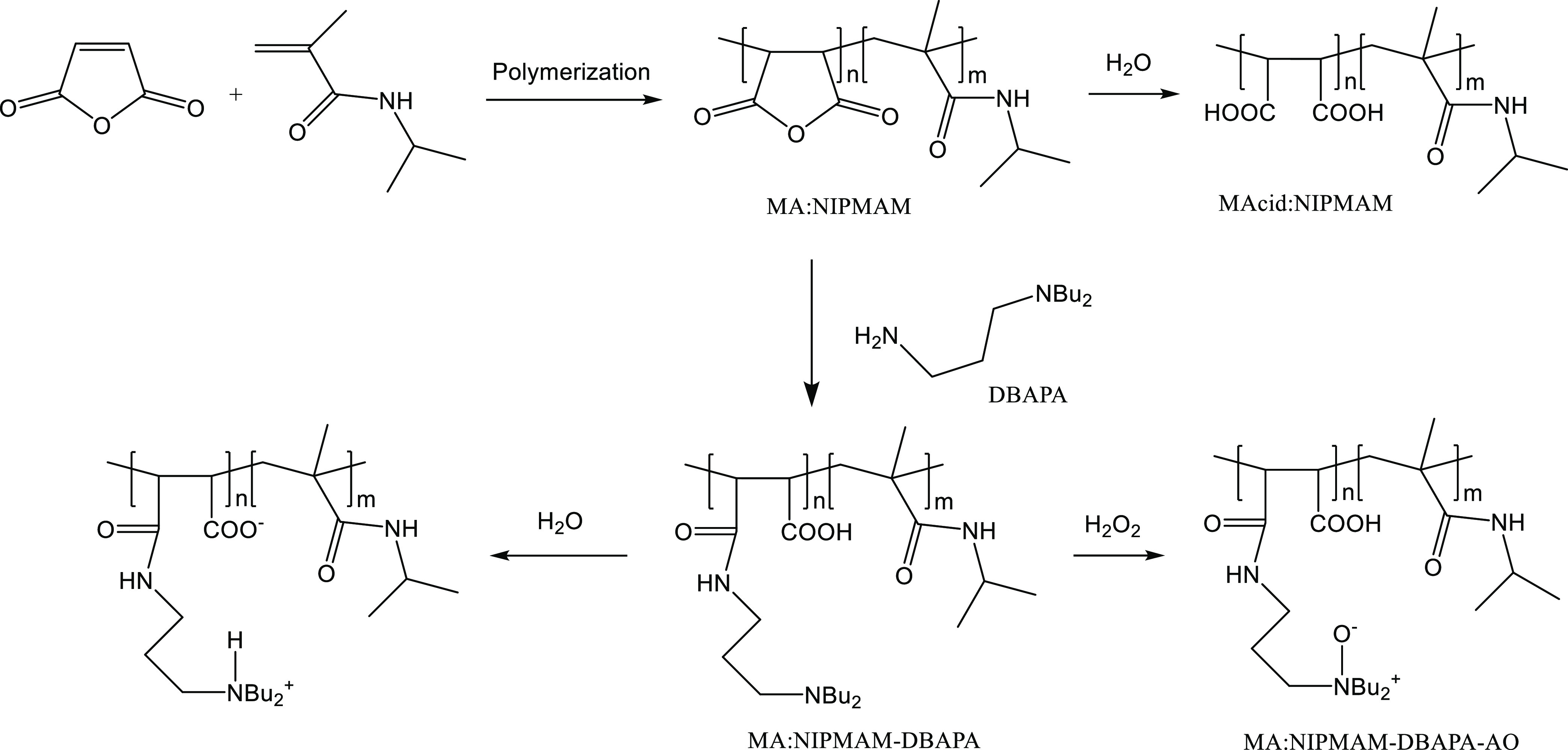
Schematic showing the synthesis of the maleic-based copolymers in this study.

The polymerization method used in this study (Figure 1) was based on an earlier published work with minor modifications.^26^ In brief, maleic anhydride (2.45 g, 0.025 mol) was copolymerized with *N*-isopropylmethacrylamide (3.18 g, 0.025 mol) using 2,2-azobis(2-methylpropionitrile) (0.41 g, 0.0025 mol) as the initiator. The reaction was carried out at 70 °C using 50 mL of 1,2-dimethoxyethane as the solvent. The reaction was carefully done in an oxygen-free atmosphere and under stirring for 15 h. After the reaction, the mixture was cooled, and the solvent was removed using rotary evaporation. The number-average molar mass (*M*_n_) of the product poly(maleic anhydride-*co*-*N*-isopropylmethacrylamide) (MA:NIPMAM) was determined using gel permeation chromatography (GPC) with dimethylformamide as the solvent and commercial polystyrenes as the standards. The solvent flow rate of the GPC was 0.6 mL/min, and the temperature was 40 °C. The MA:NIPMAM was then further modified by reacting the maleic groups with 3-(dibutylamino)-1-propylamine (DBAPA) in 2-butoxyethanol solvent to produce a DBAPA-modified poly(maleic anhydride-*co*-*N*-isopropylmethacrylamide) (MA:NIPMAM-DBAPA).^26^ Further modification of the amine groups of MA:NIPMAM-DBAPA with hydrogen peroxide produced MA:NIPMAM-DBAPA-AO (Figure 1).^26^ The MA:NIPMAM copolymer was tested for the gas hydrate inhibition effect by dissolving it in water to produce MAcid:NIPMAM (Figure 1). The MA:NIPMAM-DBAPA and MA:NIPMAM-DBAPA-AO were also tested in a similar manner but without removing 2-butoxyethanol because the solvent slightly improves the KHI performance when added to maleic-based polymers.^24^

### Gas Hydrate Inhibition Testing

The gas hydrate inhibition testing procedure in this study was based on the standard slow constant cooling (SCC) test developed by our research group.^15–26^ The instrument used was the rocking equipment RC5 as supplied by PSL Systemtechnik (Germany). The RC5 was equipped with a temperature controller and pressure/temperature sensors. Five 40 mL stainless steel cells manufactured by Safwas (Norway) were used in the RC5 equipment. Each of the cells contained one stainless steel ball, which was used for agitation. The synthetic natural gas (SNG) used in this study was based on a composition found in Table 1. The SNG forms structure II gas hydrates as the most thermodynamically stable phase at high pressures and low temperatures.^6^

**Table 1:** Synthetic Natural Gas (SNG) Used in This Study

component	mol %
methane	80.4
ethane	10.3
propane	5.00
CO_2_	1.82
isobutane	1.65
n-butane	0.72
nitrogen	0.11

The following method is a summary of the SCC test employed in this study:

1. Desired concentrations of test chemicals in deionized water were prepared a day before starting the test.
2. 20 mL samples of the test solutions were added into each cell.
3. The air in the cells was vacuumed and then purged with SNG and then pressurized to a final pressure of 76 bar.
4. The cells were programmed to rock at 20 rocks per minute at an angle of 40°.
5. The cells were then programmed to cool at 1.0 °C/h from a starting temperature of 20.5 °C down to 2.0 °C.

**Figure 2:**
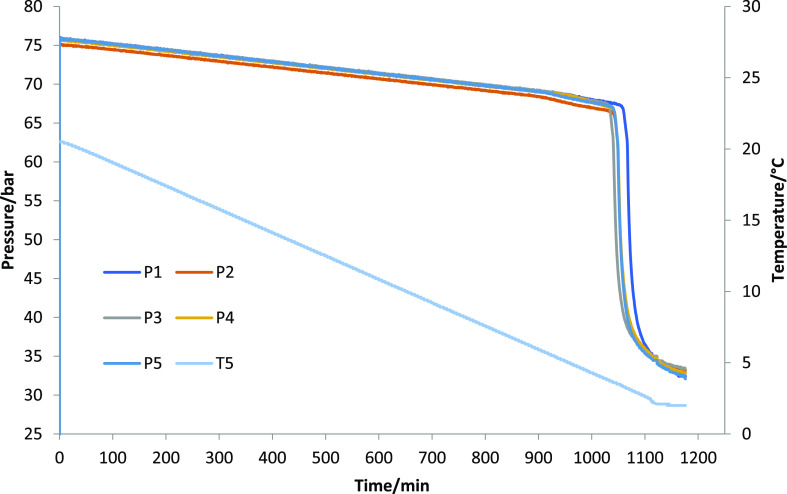
Typical graph of an SCC test using five cells. The temperature (T5) is shown decreasing constantly from 20.5 to 2 °C.

**Figure 3:**
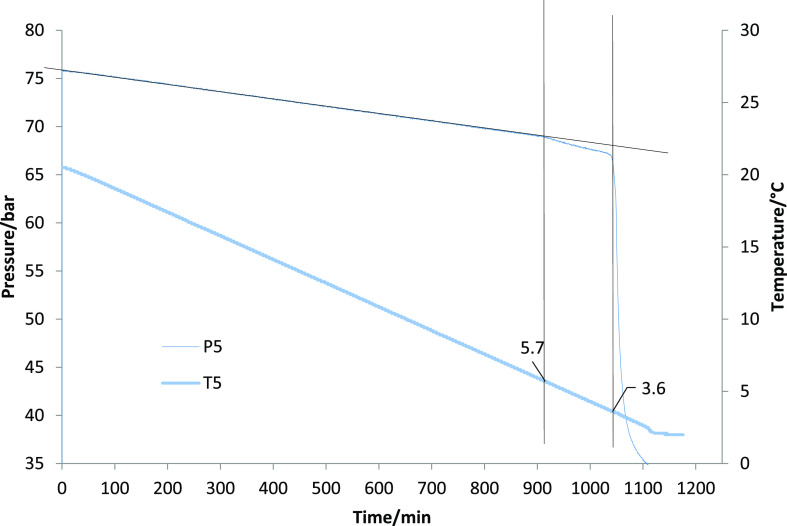
Sample determination of *T*_o_ and *T*_a_ for one SCC test.

Dissociation experiments were carried out to determine the gas hydrate equilibrium temperature (*T*_eq_), and then, the results were compared to the calculations predicted by PVTsim software (Calsep, Denmark). The *T*_eq_ at 76 bar was shown to be 20.2 ± 0.05 °C, with a 0.025 °C/h warming for the last 3–4 °C.^30^ During the course of the SCC test as described above, the pressure decreased linearly with a decrease in the temperature. When the gas hydrates started to form, however, the pressure dropped more rapidly because the gas molecules in the steel cell become trapped inside the water cages. The temperature at which this occurs was labeled as the onset temperature (*T*_o_). The temperature for the first observation of the steepest pressure drop was labeled as *T*_a_. Thus, at this temperature, the rate of the gas hydrate formation was faster than any prior temperature. Figure 2 shows a typical graph of an SCC test showing the pressure curves of all five cells. For clarity, only the temperature curve of one cell, cell 5, is shown. Figure 3 shows the actual determination of the *T*_o_ and *T*_a_ values. Both figures are representative graphs for a 5000 ppm of MA:NIPMAM-DBAPA test solution. *T*_o_ is the most important value for screening the KHIs for performance ranking. The *T*_a_ value can give some indication of the ability of KHI to slow the hydrate crystal growth. However, the subcooling at onset of crystal growth should be similar when comparing KHIs. The standard deviation (assuming a normal distribution) for a set of *T*_0_ or *T*_a_ values is no more than 0.6 °C and is usually less than 0.3 °C. The scattering still allows for a rough ranking of the performance of the KHI samples as long as sufficient tests are carried out for a statistically significant difference using a *t*-test. Depending on the variation in average *T*_0_ between the samples, 5–10 tests as done in this study are usually sufficient to get a significant difference at the 95% confidence level (*p* < 0.05).^31^

### Corrosion Inhibition Testing

**Figure 4:**
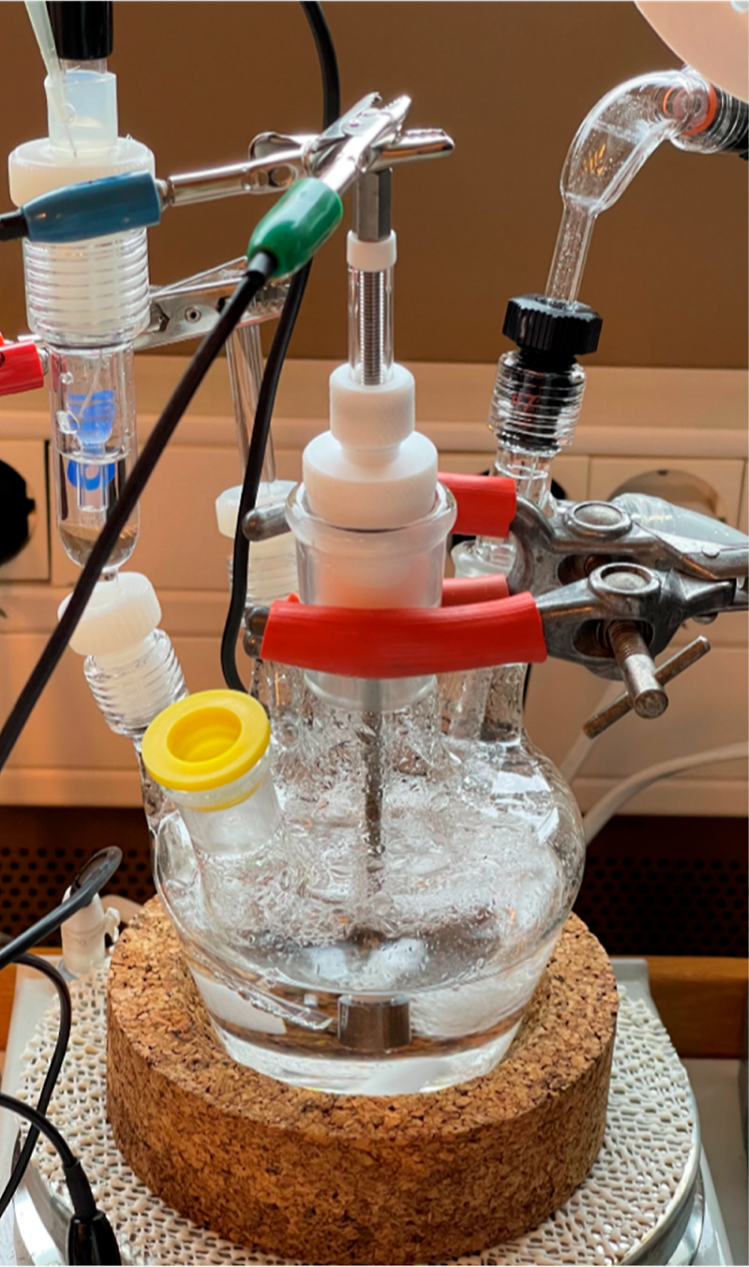
Experimental setup for CO_2_ corrosion inhibition testing.

The procedure for testing the potential CIs was based on our previous publication.^28^ The instrument used was a EuroCell Electrochemical Cell Kit from Gamry Instruments (Pennsylvania, USA). Figure 4 shows a typical setup of the corrosion testing apparatus. The apparatus was a three-electrode system with a working electrode that could hold a 5 cm^2^ C1018 metal coupon made of mild steel, a counter electrode made of Pt metal, and a reference electrode. The reference electrode in this case was an Ag/AgCl electrode that was saturated with KCl with an electrode potential of 199 mV vs normal hydrogen electrode (NHE).

In the test, high-purity (<10 ppb O_2_) CO_2_ gas was used to bubble through a solution of 80–100 mL of 3.6% aqueous NaCl. The CO_2_ gas was directly introduced into the solution using a gas dispersion tube while the solution was stirred at 250–300 rpm. This was carried out for at least 30 min prior to the test while ensuring a high/continuous CO_2_ gas flow and a solution pH of 4–5. Shortly before starting the test, the stirring rate was lowered to 200 rpm. The linear polarization resistance (LPR) measurements were then performed by introducing a ±20 mV potential to the steel relative to its open-circuit potential (*E*_oc_). Baseline tests without inhibitors were carried out for 1–2 h, ensuring a corrosion rate of around 1.8–2.1 mmpy. The test sample was then added using a syringe, and the LPR measurements were then carried out. To calculate the percent inhibition efficiency (% η), the following formula was used
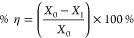
1

*X*_0_ designates the average corrosion rate without the test sample (baseline), and *X*_1_ refers to the corrosion rate with the added test sample. In this study, corrosion inhibition results were considered good if the inhibition efficiency reached at least 90%.^28^

## Results and Discussion

### Gas Hydrate Inhibition of Polymers

**Figure 5:**
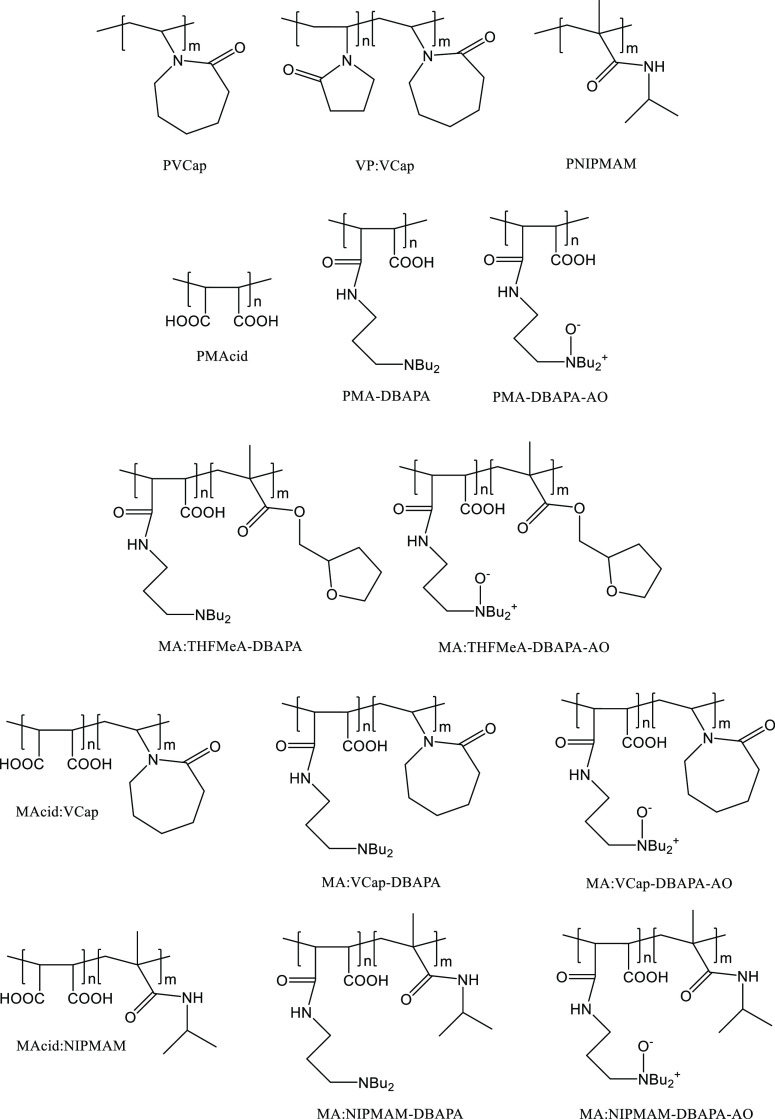
Chemical structures of different commercial polymers and maleic-based polymers.

Figure 5 shows the chemical structures of the different polymers that are relevant to this study. It includes three commercial classic gas hydrate inhibitors, such as poly(*N*-vinylcaprolactam) (PVCap), poly(*N*-vinyl-2-pyrrolidone-*co*-*N*-vinylcaprolactam) (VP:VCap), and poly(*N*-isopropylmethacrylamide) (PNIPMAM). The figure also shows the relevant homo- and copolymers of maleic anhydride that were previously synthesized by our research group.^25–27^

**Figure 6:**
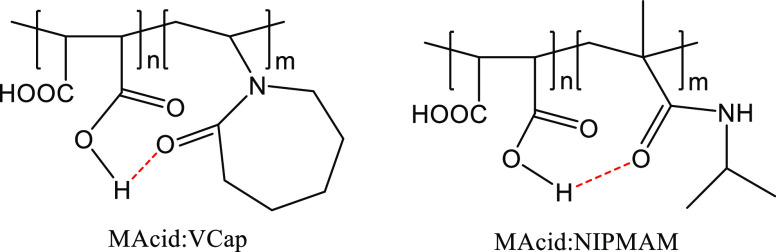
Intramolecular hydrogen bonding of MAcid:VCap and MAcid:NIPMAM polymers in aqueous solution.

The gas hydrate inhibition effects of the above-mentioned polymers are summarized in Table 2. It is also organized by composition, as in Figure 5. Underivatized poly(maleic anhydride-*co*-tetrahydrofurfuryl methacrylate) was not included because this polymer was insoluble in water.^25^ A closer look at Table 2 reveals some key trends. First, the underivatized MAcid:NIPMAM copolymer showed mediocre gas hydrate inhibition performance with a *T*_o_/*T*_a_ of 12.0/11.9. This result was analogous to the mediocre performance of the underivatized MAcid:VCap in our previous publication. We were able to computationally demonstrate that the poor performance of MAcid:VCap could be attributed to the intramolecular hydrogen bonding that caused the polymer chain to form a globular conformation, which subsequently reduced the exposure of the active caprolactam ring.^26^ In principle, this could also be applied to the MAcid:NIPMAM polymers in this study. Figure 6 shows the possible intramolecular hydrogen bonding that might have occurred. The MAcid:NIPMAM polymer could presumably form a globular conformation, which also hid the active amide functional group.

**Table 2:** Gas Hydrate Inhibition Performance in SCC Tests for 2500 ppm of Comparative Commercial Polymers and Maleic-Based Polymers

polymer name	Mn g/mol	*T*_o_ (av.) [°C]	St. Dev. [°C]	*T*_a_ (av.) [°C]
no polymer		17.1	0.5	16.9
PVCap	8000	9.7	0.3	9.4
VP:VCap	2000–4000	8.5	0.5	6.4
PNIPMAM^28^	22,400	9.3	0.7	9.0
PMAcid	800	16.1	0.6	15.9
PMA-DBAPA	2300	9.5	0.4	9.4
PMA-DBAPA-AO	2500	8.7	0.5	8.3
MA:THFMeA-DBAPA	3900	6.9	0.3	6.1
MA:THFMeA-DBAPA-AO	4030	6.5	0.2	6.3
MAcid:VCap	15,500	14.2	0.5	13.9
MA:VCap-DBAPA	16,600	5.6	0.4	5.4
MA:VCap-DBAPA-AO	17,300	3.9–5.2	0.4	3.7–4.5
MAcid:NIPMAM	18,900	12.0	0.6	11.9
MA:NIPMAM-DBAPA	33,800	6.5	1.1	5.9
MA:NIPMAM-DBAPA-AO	35,200	6.0	1.1	5.6

The second observation in Table 2 is that the maleic-based copolymers generally perform better compared with the corresponding maleic-based homopolymers. A detailed discussion of the gas hydrate inhibition performance of our maleic-based homopolymers is found in our previous publication.^27^ PMA-DBAPA showed a gas hydrate inhibition performance that is comparable to that of the classic PVCap and PNIPMAM polymers. However, both the copolymers MA:VCap-DBAPA and MA:NIPMAM-DBAPA performed better in comparison to PMA-DBAPA, PVCap, or PNIPMAM. This could suggest that the presence of VCap or NIPMAM units together with the PMA-DBAPA units within the same macromolecule caused a synergistic inhibitory effect.

**Figure 7:**
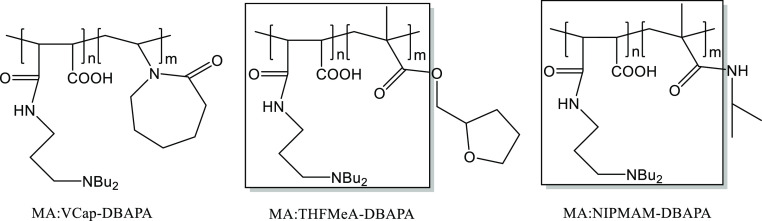
Structural comparison of MA:VCap-DBAPA, MA:THFMeA-DBAPA, and MA:NIPMAM-DBAPA.

Table 2 shows that both the copolymers MA:THFMeA-DBAPA and MA:NIPMAM-DBAPA gave similar gas hydrate inhibition performance with *T*_o_/*T*_a_ values of 6.9/6.1 °C and 6.5/5.9 °C, respectively. Figure 7 shows a comparison between the structures of MA:THFMeA-DBAPA and MA:NIPMAM-DBAPA as well as MA:VCap-DBAPA. Both MA:THFMeA-DBAPA and MA:NIPMAM-DBAPA copolymers contain a common structural motif, which may explain why they have similar *T*_o_/*T*_a_ values. In contrast, MA:VCap-DBAPA is a structurally different relative to the other two, which may explain why it has different *T*_o_/*T*_a_ values.

Table 3 shows the effects of polymer concentration on the gas hydrate inhibition performance of the MA:NIPMAM-DBAPA or MA:NIPMAM-DBAPA-AO polymers. For comparison, the results for VP:VCap, MA:VCap-DBAPA, and MA:VCap-DBAPA-AO from our previous study are also shown.^28^ The trend shows that the performance of MA:NIPMAM-DBAPA or MA:NIPMAM-DBAPA-AO improved as the concentration increased. In our previous paper, we hypothesized that MA:VCap-DBAPA or MA:VCap-DBAPA-AO might aggregate in solution at 5000 ppm, which explained the decrease in performance at that concentration.^28^ In this study, however, micellization of MA:NIPMAM-DBAPA or MA:NIPMAM-DBAPA-AO might not have occurred, which explains the improved performance at increasing concentrations. This makes field applications easier to manage because the chemical can be deployed at increasing concentrations without worrying about the loss of performance.

**Table 3:** Effects of Concentration on the Gas Hydrate Inhibition Performance (the Values are Average *T*_o_/*T*_a_ in °C)

polymer	concentration/ppm			
	0	1000	2500	5000
VP:VCap	17.1/16.9	10.5	8.5/6.3	6.4/4.2
MA:VCap-DBAPA		8.8/8.0	4.8/4.5	5.6/4.0
MA:VCap-DBAPA-AO		8.4/7.7	4.3/3.7	5.2/3.3
MA:NIPMAM-DBAPA		8.5/8.0	6.5/5.9	5.3/3.6
MA:NIPMAM-DBAPA-AO		8.0/7.7	6.0/5.6	4.4/3.1

### Gas Hydrate Inhibition of Polymers with Synergists

Within the context of this study, we define synergists as chemicals added in relatively smaller amounts to our MA:NIPMAM-DBAPA or MA:NIPMAM-DBAPA-AO copolymers to boost the performance. The synergists do not necessarily have gas hydrate inhibition capabilities on their own but enhance the overall gas hydrate inhibition performance together with the main polymer inhibitor. More than one mechanism of synergism may be taking place reducing the rate of hydrated nucleation and/or crystal growth.^28^

For our studies, an ideal KHI synergist would also enhance the overall corrosion inhibition performance. From a structural standpoint, we envision a candidate CI synergist to enhance the maleic-based polymer CI effect by containing other electron-rich functional groups that bind to Fe on the steel surface. The classical functional groups that are used in corrosion inhibition usually contain nitrogen, sulfur, and sometimes phosphorus or oxygen.^6^ We therefore judiciously chose chemicals that contain these heteroatoms as candidates for synergists in this study. In addition, some synergists contained butyl groups since some butylated molecules are known to be good KHI synergists such as *n*- or iso- butyl glycol ethers.^32–37^

**Figure 8:**
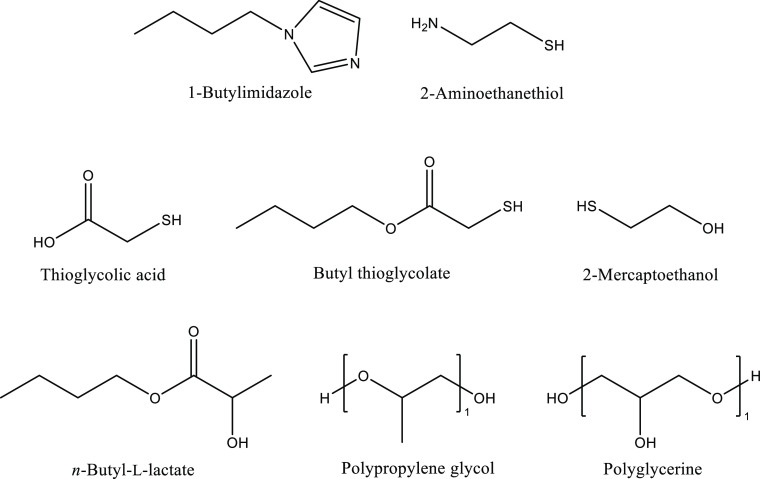
Chemical structures of the synergists in this study.

Figure 8 shows the structures of the chemicals that were first tested for potential KHI synergism. These are arranged into common structural features. The nitrogen-containing compounds (1-butylimidazole and 2-aminoethanethiol, the latter of which also contains sulfur) are shown in the first row. The second row shows the sulfur-containing chemicals (thioglycolic acid, butyl thioglycolate, and 2-mercaptoethanol). Lastly, the third row shows the oxygen-containing chemicals (*n*-butyl-l-lactate, poly(propylene glycol), and polyglycerine).

Tables 4 and 5, respectively, show the gas hydrate inhibition results of 2500 ppm of MA:NIPMAM-DBAPA-AO and that of MA:NIPMAM-DBAPA with added 1000 ppm of synergists. This amount of synergist may be higher than that needed for optimal corrosion inhibition, but we wanted to add sufficient synergy to enable us to observe if there was KHI synergy. Chemicals that have poor synergistic effect with MA:NIPMAM-DBAPA-AO (*T*_o_ lowering of 0.3 °C and below) were not tested again with MA:NIPMAM-DBAPA. As the tabulated results indicate, the best synergist that gave the lowest overall decrease in average *T*_o_/*T*_a_ was butyl thioglycolate. It had good synergistic effects with both MA:NIPMAM-DBAPA and MA:NIPMAM-DBAPA-AO, lowering the average *T*_o_ to 2.4 and 1.8 °C, respectively. These are statistically significant results (*t*-test: *p* < 0.05). Butyl thioglycolate, however, did not show a gas hydrate inhibition effect on its own. 2500 ppm of butyl thioglycolate gave an average *T*_o_/*T*_a_ of 16.9/16.0 °C, comparable to the *T*_o_/*T*_a_ of pure water. Since other butylated molecules such as 1-butylimidazole did not show comparable synergy, we propose that the synergetic activity of butyl thioglycolate requires chemical interactions with MA:NIPMAM-DBAPA or MA:NIPMAM-DBAPA-AO, possibly between the thiol and amine groups. The interaction might also alter the polymer conformation and subsequently expose the hydrate-inhibiting functional groups of MA:NIPMAM-DBAPA or MA:NIPMAM-DBAPA-AO. We have shown in our previous research that macromolecular conformations can have drastic effects on a polymer’s gas hydrate inhibition performance.^26^

**Table 4:** Gas Hydrate Inhibition Results for 2500 ppm of MA:NIPMAM-DBAPA-AO with 1000 ppm of Synergists

synergist	*T*_o_ (av.) [°C]	St. Dev. [°C]	*T*_a_ (av.) [°C]
MA:NIPMAM-DBAPA-AO only	6.0	1.1	5.6
1-butylimidazole	5.3	0.7	4.8
2-aminoethanethiol	5.7	0.4	4.6
thioglycolic acid	6.5	0.6	5.7
butyl thioglycolate	4.2	0.5	3.6
2-mercaptoethanol	5.8	2.0	4.3
n-butyl-l-lactate	5.2	0.6	4.9
polypropylene glycol 400, *M*_w_ 400 g/mol	5.5	0.8	4.9
polyglycerine, *M*_w_ 1000 g/mol	5.8	0.3	5.4

**Table 5:** Gas Hydrate Inhibition Results for 2500 ppm MA:NIPMAM-DBAPA with 1000 ppm Synergists

synergist	*T*_o_ (av.) [°C]	St. Dev. [°C]	*T*_a_ (av.) [°C]
MA:NIPMAM-DBAPA only	6.5	1.1	5.9
1-butylimidazole	5.4	0.3	5.0
butyl thioglycolate	4.1	0.2	4.0
n-butyl-l-lactate	5.7	0.4	5.2
polypropylene glycol 400, *M*_w_ 400 g/mol	5.6	0.1	5.2

It is also interesting to note that thioglycolic acid (an analogue of butyl thioglycolate) does not show a synergistic effect with MA:NIPMAM-DBAPA-AO. In fact, thioglycolic acid increased the *T*_o_ of MA:NIPMAM-DBAPA-AO by 0.5 °C although this is not a statistically significant increase [*t*-test: (*p* > 0.05)]. We propose that the butyl chain in butyl thioglycolate plays a role in the synergistic effect as it could also help disrupt the formation of gas hydrate cages. In fact, two of the other chemicals that contain a butyl group (1-butylimidazole and *n*-butyl-l-lactate) had possible mild synergistic effects with MA:NIPMAM-DBAPA-AO, lowering *T*_o_ by 0.7 and 0.8 °C, respectively, although the results are not statistically significant (*t*-test: *p* > 0.05).

**Figure 9:**
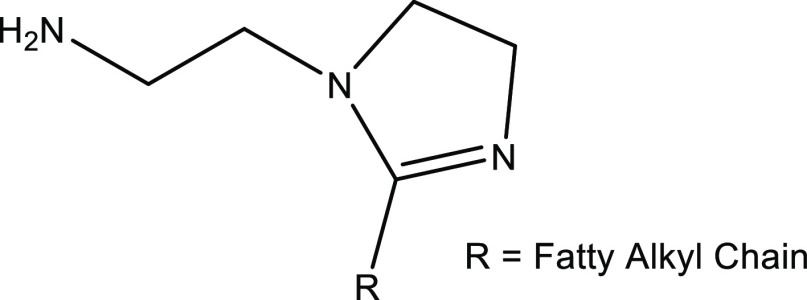
Structure of fatty acid imidazoline.^40^

For comparison purposes, we also tested the commercial fatty acid imidazoline (Figure 9). This is a film-forming chemical and a well-known antagonist of some KHI polymers.^6,28,38,39^ Fatty acid imidazoline was tested either alone or in combination with other chemicals in this study (a summary is shown in Table 6). The results revealed that the fatty acid imidazoline itself did not have a kinetic hydrate inhibition effect on its own. However, it reduced the overall performance of PVCap from an average *T*_o_ of 9.7 to 11.9 °C. Interestingly, both MA:NIPMAM-DBAPA and MA:NIPMAM-DBAPA-AO seem to be resistant to this type of antagonism since the *T*_o_/*T*_a_ remained statistically unchanged before and after the addition of fatty acid imidazoline. This demonstrates that fatty acid imidazoline is indeed incompatible with the classic PVCap and not with the MA:NIPMAM-DBAPA or MA:NIPMAM-DBAPA-AO copolymers in this study.

**Table 6:** Summary of the Antagonistic Effect of Commercial Fatty Acid Imidazoline^28^

additive name	*T*_o_ (av.) [°C]	St. Dev. [°C]	*T*_a_ (av.) [°C]
none	17.1	0.5	16.9
2500 ppm PVCap	9.7	0.3	9.4
2500 ppm fatty acid imidazoline	17.6	0.4	17.5
2500 ppm PVCap + 500 ppm fatty acid imidazoline	11.9	0.4	9.0
2500 ppm MA:NIPMAM-DBAPA-AO	6.0	1.1	5.6
2500 ppm MA:NIPMAM-DBAPA-AO + 500 ppm fatty acid imidazoline	6.1	1.4	4.4
2500 ppm MA:NIPMAM-DBAPA	6.5	1.1	5.9
2500 ppm MA:NIPMAM-DBAPA + 500 ppm fatty acid imidazoline	6.1	1.6	4.0

### Corrosion Inhibition Results

Table 7 shows the results of the corrosion inhibition testsperformed in this study. Due to the insolubility of MA:NIPMAM-DBAPA-AO in the presence of 3.6% NaCl aqueous solution, only MA:NIPMAM-DBAPA was further tested for corrosion inhibition. The incompatibility of MA:NIPMAM-DBAPA-AO with brine was surprising considering that MA:VCap-DBAPA-AO was fully soluble at the same conditions.^28^ The results in Table 7 show that 500 ppm MA:NIPMAM-DBAPA without added synergists made sweet corrosion worse (−34.7% efficiency). A negative result was also observed for 2500 ppm of DBAPA-modified poly(maleic anhydride-*co*-*N*-vinylcaprolactam) (MA:VCap-DBAPA), which caused −75.0% inhibition efficiency under similar conditions. MA:VCap-DBAPA at 500 ppm, however, gave a small corrosion inhibition efficiency of 18.1%.^28^ This observation might come as a surprise since MA:NIPMAM-DBAPA has many nitrogen-containing functional groups in the macromolecule, which, in theory, should suppress corrosion.^28^ We speculate, however, that these nitrogen atoms were quaternized, which means that no electron lone pairs were available for interaction with empty iron d-orbitals for chemisorption at the metal surface. This effectively isolates the metal from the corrosive aqueous environment.^41,42^

**Table 7:** Corrosion Inhibition Testing Results for MA:NIPMAM-DBAPA with Synergists

main additive	synergist	concentration, ppm	inhibition efficiency %	S.D. %
MA:NIPMAM-DBAPA		500	–34.7	24
	fatty acid imidazoline	500 + 100	77.6	25
	2-aminoethanethiol	500 + 100	97.4	6.1
	butyl thioglycolate	500 + 100	96.9	6.6
	2-mercaptoethanol	500 + 100	97.3	8.9
	thioglycolic acid	500 + 100	98.4	5.6
fatty acid imidazoline		100	76.5	5
butyl thioglycolate		100	94.2	

Table 7 also shows the corrosion inhibition efficiency of MA:NIPMAM-DBAPA in combination with other chemicals. The compatibility of commercial fatty acid imidazoline with MA:NIPMAM-DBAPA was first assessed. Fatty acid imidazoline on its own at 100 ppm had a corrosion inhibition efficiency of around 76.5%. When combined with 500 ppm of MA:NIPMAM-DBAPA, however, the overall corrosion inhibition efficiency was 77.6%. This indicates that MA:NIPMAM-DBAPA did not significantly affect the performance of fatty acid imidazoline. Thus, MA:NIPMAM-DBAPA and fatty acid imidazoline were found to be compatible from both hydrate and corrosion inhibition objectives.

The best-performing KHI synergist in Table 5 (butyl thioglycolate) was tested for corrosion inhibition in combination with MA:NIPMAM-DBAPA. It was initially planned to test 2500 ppm of MA:NIPMAM-DBAPA with 1000 ppm of butyl thioglycolate, as in Table 5. The MA:NIPMAM-DBAPA solution, however, caused considerable foaming with the CO_2_ bubbling. A high concentration of butyl thioglycolate such as this can also cause overloading as in our previous publication and is not necessary for optimum CI effect.^28^ We therefore opted to test 500 ppm of MA:NIPMAM-DBAPA and 100 ppm of butyl thioglycolate. The results in Table 7 show that this combination showed good corrosion inhibition of about 96.9%. This result is better than butyl thioglycolate on its own (94.2%), i.e., the combination of the two chemicals roughly halves the corrosion rate of using just 100 ppm of butyl thioglycolate. Since the polymer by itself does not give good corrosion protection, we tentatively suggest that there may be some interaction of the polymer and butyl thioglycolate that gives better chemisorption to the steel surface than that with either chemical alone. To compare to our previous publication,^28^ we also tested other thiolated products, 2-aminoethanethiol, 2-mercaptoethanol, and thioglycolic acid, at the same concentration. The results show that these chemicals also enhanced the overall corrosion inhibition efficiency of MA:NIPMAM-DBAPA with values that reached around 97–98%. However, these molecules are, at best, only very mild KHI synergists. Both MA:NIPMAM-DBAPA and MA:VCap-DBAPA, therefore, need to be combined with thiol-containing compounds to achieve good corrosion inhibition results. It is known, however, that in order for the formulation to also achieve good performance as a KHI, the added molecule must also contain a short alkyl chain, the same size as alkane gas hydrate formers.^28^ In this case, the butyl chain of butyl thioglycolate might also help in the disruption of gas hydrate formation, which explains its good synergistic effect shown in Table 5.

## Conclusions

Poly(maleic anhydride-*co*-*N*-isopropylmethacrylamide) (MA:NIPMAM) and its derivatives for potential simultaneous gas hydrate and corrosion inhibition were successfully synthesized. Underivatized polymer in water (MAcid:NIPMAM) showed poor gas hydrate inhibition performance, possibly due to internal hydrogen bonding. Derivatization of MA:NIPMAM with 3-(dibutylamino)-1-propylamine (DBAPA) gave MA:NIPMAM-DBAPA, which was easily converted to amine oxide MA:NIPMAM-DBAPA-AO. MA:NIPMAM-DBAPA was compatible with a film-forming fatty acid imidazoline CI in that the KHI and CI performances were not affected adversely. Blending small thiol-based molecules to MA:NIPMAM-DBAPA greatly enhanced the CI performance better than the fatty imidazoline. One of these thiols, butyl thioglycolate, was also able to enhance the KHI performance of MA:NIPMAM-DBAPA. Other butylated molecules did not show this KHI-enhancing effect, which suggests that some cooperative action between the KHI polymer and butyl thioglycolate is occurring.^43^ This may also be one of the reasons for the good corrosion inhibition of the MA:NIPMAM-DBAPA + butyl thioglycolate mixture.
